# Urbanization increases stochasticity and reduces the ecological stability of microbial communities in amphibian hosts

**DOI:** 10.3389/fmicb.2022.1108662

**Published:** 2023-01-12

**Authors:** Jin Zhou, Ziyan Liao, Zhidong Liu, Xuecheng Guo, Wenyan Zhang, Youhua Chen

**Affiliations:** ^1^China-Croatia “Belt and Road” Joint Laboratory on Biodiversity and Ecosystem Services, Chengdu Institute of Biology, Chinese Academy of Sciences, Chengdu, China; ^2^Key Laboratory of Bio-Resources and Eco-Environment of Ministry of Education, College of Life Sciences, Sichuan University, Chengdu, Sichuan, China; ^3^University of Chinese Academy of Sciences, Beijing, China

**Keywords:** biotic homogenization, disturbance, abiotic gradient, community patterns, ecological niches

## Abstract

Urbanization not only profoundly alters landscape profiles, ecosystems and vertebrate faunal diversity but also disturbs microbial communities by increasing stochasticity, vulnerability, biotic homogenization, etc. However, because of the buffering effect of host species, microbial communities are expected to be influenced by both host species and urbanization stresses. Therefore, the impacts of urbanization on animals’ microbial symbionts could be more complex and uncertain. In this study, we quantified the urbanization degree of sampling sites and surveyed the gut and skin microbes of three amphibian host species in different sites in urban parks and nearby villages of Chengdu, Southwest China. Furthermore, a co-occurrence network analysis, the phylogenetic normalized stochasticity ratio and Sloan neutral community models were applied to infer the impact of urbanization on symbiotic microbial communities. For the three host species, urbanization increased the diversity of symbiotic microbes and the number of keystone microbial taxa. However, the negative effects of such increased diversification were evident, as the community stochasticity and co-occurrence network structure vulnerability also increased, while the network structure complexity and stability were reduced. Finally, the community stochasticity had positive associations with the network vulnerability, implying that the existence of many transient symbiotic rare microbial taxa in urban parks makes the symbiotic microbial community structure more fragile. Conclusively, urbanization increased the symbiotic microbial diversity at the cost of community stability; the results provide a new perspective for better understanding the complex triangulated environment–host–microbe relationship.

## 1. Introduction

Urbanization is one of the most direct manifestations of human activities (Rothwell et al., 2015; Zhai et al., 2020) and has been a major cause of the biodiversity crisis, which has remarkably reduced the suitable habitats available for wild animals to survive and breed (Grimm et al., 2008; McDonald et al., 2008; Fenoglio et al., 2020; Li et al., 2022). Various studies have reported the effects of urbanization on macroecological biodiversity patterns (Kondratyeva et al., 2020; Li et al., 2022; Pan et al., 2022). However, few studies (Lokmer et al., 2020; Barnes et al., 2021) have reported the impacts of urbanization on the biological diversity and composition of microorganisms associated with the organs of animal hosts.

Cities are expected to expand due to increase in the population through immigration from neighboring wild areas and the aggregation of supporting infrastructure, particularly in developing countries (Henderson, 2002; Xu et al., 2014; Zhai et al., 2020). Therefore, urbanization is inevitable and will be intensified. Over the past decade, Chengdu has been the fastest-growing city in Southwest China, with a total population of over 20 million. Consequently, suitable habitats for wild animals to inhabit within city boundaries have been greatly reduced (Kong et al., 2010). Furthermore, as a result of increased urbanization, these ‘green spaces’ are continuously fragmented and isolated, leaving wild animals vulnerable to human disturbance (Gaston, 2010) (e.g., pollution, roadkill, and illegal injury) and making it less likely to avoid population decline or local extinction through migration (Marzluff and Rodewald, 2012; Bendik et al., 2014). This equally applies to microorganisms of host species. Because of the difficulty of finding suitable habitats for animal host species in cities, microbial communities of the hosts are also greatly selected and modulated by urban environments in comparison to those in rural and wild habitats (Lokmer et al., 2020; Barnes et al., 2021).

Over one-third of amphibian species are threatened by urbanization (Hamer and McDonnell, 2008), which may be because amphibians are the most sensitive taxa in response to climate and urban environments compared with others such as birds and mammals (Hamer and McDonnell, 2008; Magle et al., 2012; Callaghan et al., 2021). Small patches and fragmented habitats caused by urbanization can remarkably affect the home range of animals and, as a result, their reproduction and survival, particularly for amphibians (Hinam and Clair, 2008; Decout et al., 2012; Amburgey et al., 2021). However, as amphibians are small-bodied animals, they can survive in small patches of forested and humid areas of ‘green spaces’, provided that these areas are not heavily polluted or disturbed (Hamer and McDonnell, 2008). The impact of urbanization on the biodiversity (Callaghan et al., 2021; Yang, L. et al., 2022) and genetic structure (Wei et al., 2021) of amphibian populations has been extensively studied, but microbial data records associated with amphibian hosts in urban settings are lacking.

**Figure 1:**
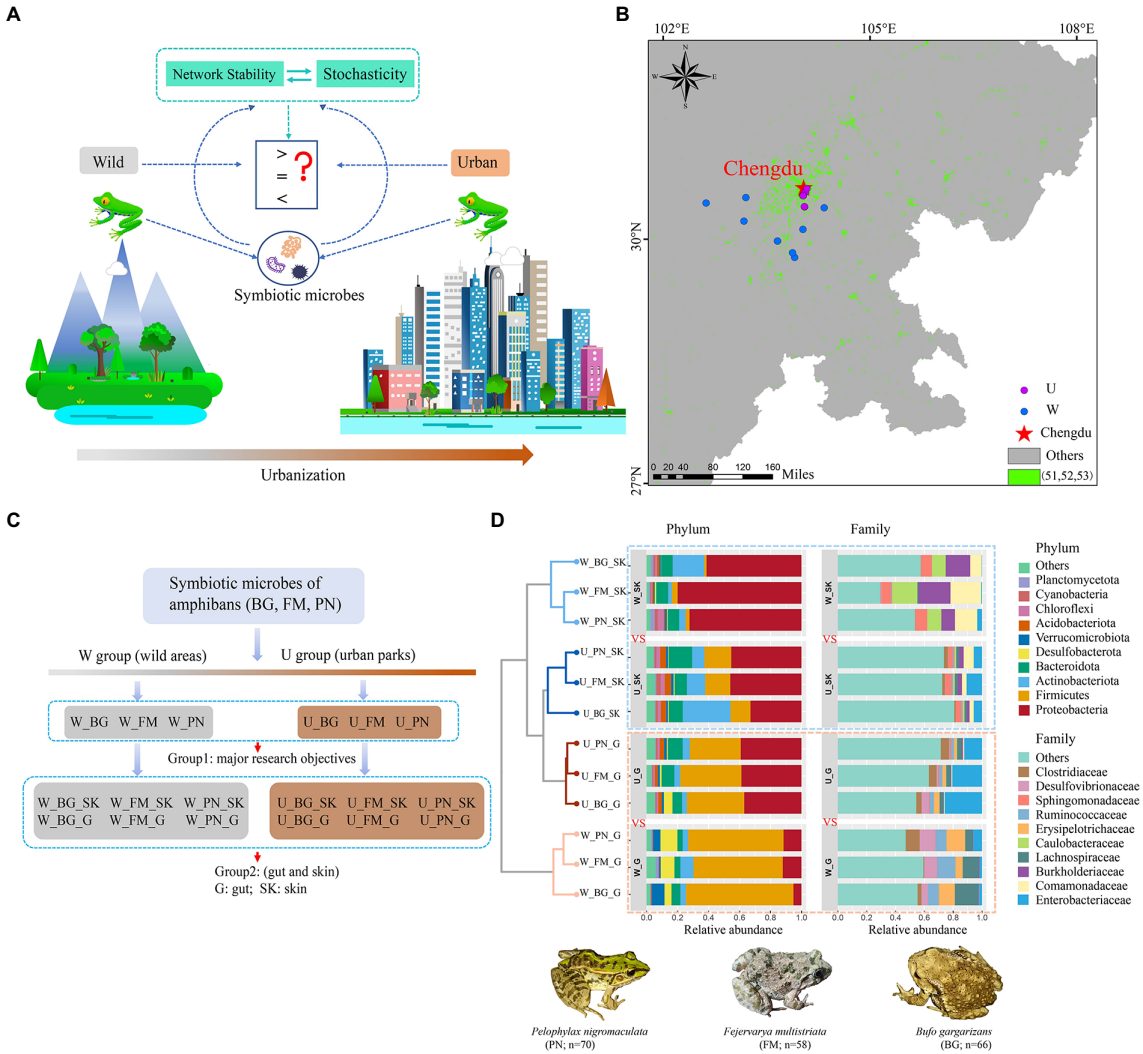
Community composition of microbes of amphibians in the urban parks and wild area habitats. **(A)** Schematic illustration of the effects of urbanization on the community network stability and assembly mechanisms of amphibian symbiotic microorganisms. **(B)** Diagram of grouping. **(C)** Map of sampling sites in city parks and wild areas of Chengdu city in China based on the land use types of the dataset. The green points represent urban land, including the 51 (urban land), 52 (rural residential areas), and 53 (other construction land) land use types. The purple and blue points represent the U (urban park samples) and W (wild area samples) groups, respectively. **(D)** Comparison of the taxonomic composition of the top 10 phyla and family between the U and W groups. BG, *Bufo gargarizans*; FM, *Fejervarya multistriata*; PN, *Pelophylax nigromaculatus*. G, gut; SK, skin; W_G, gut microbiota of amphibians in wild areas; W_SK, skin microbiota of amphibians in wild areas; U_G, gut microbiota of amphibians in urban parks; U_SK, skin microbiota of amphibians in urban parks.

Symbiotic microorganisms are critical for amphibian survival (Becker et al., 2015) and adaptation to rapid environmental changes (Xu et al., 2020b; Barnes et al., 2021). Consequently, it is crucial to elucidate the response of the amphibian symbiotic microbial community to rapid urbanization, which is valuable for wildlife conservation in urban settings (Wei et al., 2021). However, urban ecosystems present distinctive microhabitat and microclimate conditions in comparison to wild regions (Zhang et al., 2022). Therefore, it is expected that the microorganism community structure and diversity of amphibian hosts must also alter and adapt to the urban environment. However, to the best of our knowledge, few studies have reported on the potential distinctive features (e.g., stochasticity and stability) of the microbial community structure of animal hosts in urban settings (see concept Figure 1A), especially for amphibians, which are indicator species for assessing urbanization’s ecological impact (Guzy et al., 2012).

The process of community assembly is generally used to uncover the formation mechanisms of microbial diversity and composition differences, which include deterministic processes based on niche theory and stochastic processes based on neutral theory (Stegen et al., 2013, 2015; Ning et al., 2020). Theories based on niches contend that species distribution is primarily controlled by the deterministic processes involve with abiotic factors (e.g., environmental factors) and biotic factors (e.g., biological interactions) (Stegen et al., 2013; Ning et al., 2020). In contrast, neutral theories view all species as ecologically equivalent, with stochastic processes largely determining species dynamics and patterns, such as birth/death, speciation/extinction, and migration (Stegen et al., 2013; Ning et al., 2020). Furthermore, ecological stability is vital for microbial communities to adapt to rapid environmental changes (Yuan et al., 2021; Liu et al., 2022). Generally, microbial network analysis is used to evaluate the interspecific interactions and ecological stability or complexity of microbial communities (Yuan et al., 2021; Liu et al., 2022).

In summary, we aimed to answer the following questions: (i) Does urbanization affect the diversity and community structure of symbiotic microbes? (ii) What is the actual influence of urbanization on the stochasticity and stability of amphibian symbiotic microbial communities? (iii) What is the relationship between the stochasticity and stability of symbiotic microbial communities under urbanization gradient? To this end, we chose three amphibian host species (*Pelophylax nigromaculatus*, abbr: PN; *Fejervarya multistriata*, abbr: FM; *Bufo gargarizans*, abbr: BG) that are widely distributed in the city parks and surrounding wild areas of Chengdu, southwest China, to elucidate the community assembly and diversity patterns of microbial taxa in the skins and guts of the host species. We compared the microbial differences between amphibians in urban and wild environments, between host species and between different organs of the host species. This work explores the urbanization-host animal-microbial relationship with the aim of providing new perspectives on bioenvironmental interactions in the context of rapid global land use change.

## 2. Materials and methods

### 2.1. Sample collection and sequencing

From April to August 2021, we collected 194 microbial samples from three frog species in downtown Chengdu city and surrounding wild areas, which included four city park sampling sites and eight wild sampling sites (that is, exurban regions) (Figure 1B; Supplementary Table S1). For each sampling site, we searched for frog species alongside ditches, brooks or ponds. We captured adult individuals by wearing sterile gloves and took the microbial samples from the skin by using cotton swabs after rinsing three times with sterile water to remove the potential transient bacteria (Lauer et al., 2007). A 2-ml aseptic centrifuge tube was used to collect gut microbial content after the frog was euthanized and dissected. After returning to the laboratory, all gut and skin samples were immediately stored at –80°C. Experimental approval was obtained from the Animal Ethical and Welfare Committee of Chengdu Institute of Biology, Chinese Academy of Sciences (permit no. CIBDWLL2022008).

Microbial DNA was extracted using the MN NucleoSpin 96 Soil kit (MACHEREY-NAGEL) according to the manufacturer’s instructions. The V3-V4 regions of the 16S rRNA gene were amplified using the universal primer pair 338F/341F and 806R/805R (Yao et al., 2019; Gutierrez-Villagomez et al., 2021). Briefly, PCR thermocycling was carried out under the following conditions: 95°C for 5 min, followed by 30 cycles at 95°C for 30 s, 50°C for 30 s, 72°C for 60 s and a final extension at 72°C for 5 min. The PCR products were further purified with e.Z.N.A. TM Cycle-Pure Kit (Omega).

The raw sequence data were processed using the QIIME2 software package (version QIIME2-2021.2) (Bolyen et al., 2019). The paired reads (2 × 250 bp paired-end mode) generated by the Illumina platforms of MiSeq and Nova6000 were demultiplexed and filtered by vsearch and quality filtered with the following quality-control criteria: sequences of length < 200 bp and average quality score < 25 were removed; no ambiguous bases were allowed. High-quality sequence reads with >97% identity were defined as operational taxonomic units (OTUs) with the vsearch cluster-features-de-novo (Rognes et al., 2016). Then, the samples were rarefied to the same sequence depth (33,187 bacterial sequences per sample). To minimize inclusion of sequencing errors, the clustered feature tables were further filtered using QIIME2 feature-table filter-features (Revise: p-min-samples 2 --p-min-frequency 10) (Zhang et al., 2020). Taxonomy assignment to the OTUs was performed using the Silva v138 database and the Naive Bayes classifier (Quast et al., 2012). Subsequently, the sequences identified as chloroplasts or mitochondria were removed. The final dataset retained 19,042 OTUs for the downstream analyses. In our study, we classified the collection of microbial samples from urban park samples and wild area samples into U and W groups, respectively (Figure 1B). We also separated the microbial samples into two groups for three host species in the analyses. Group 1 contained all the microbes from all the organs (gut and skin) of the three host species, including W_BG, U_BG, W_FM, U_FM, W_PN, and U_PN (Figure 1C). Group 2 distinguished the microbial communities from the skin (abbr: SK) and gut (abbr: G) ecosystems, including W_BG_G, W_BG_SK, U_BG_G, U_BG_G _SK, W_FM_G, W_FM_SK, U_FM_G, U_FM_SK, W_PN_G, W_PN_SK, U_PN_G, and U_PN_SK (Figure 1B). In this study, Group 1 was the primary research target. The analyses were conducted and compared at the OTU, family and phylum levels.

### 2.2. Measures of diversity and community structure

All result analysis and visualization were performed in R v4.1.3.^1^ For the alpha diversity, we computed the richness, Shannon index and phylogenetic diversity for each community and used the Wilcoxon rank-sum test and the false discovery rate (FDR) corrected method to identify significant differences between the U and W groups.

For the beta diversity, we compared the difference in beta diversity built on the Bray–Curtis distance among the six groups by using Kruskal–Wallis tests with multiple testing correction Behrens–Fisher using the *nmpc* function in the ‘nmpc’ R package (Munzel and Hothorn, 2001). Additionally, PERMANOVA and ANOSIM tests (number of permutations: 999) based on the Bray–Curtis distance were used to analyze the differences in gut and skin microbes between the Group U and Group W amphibians. Nonmetric multidimensional scaling (NMDS) was used to visualize the results using the ‘vegan’ R package (Oksanen et al., 2002).

We assessed whether neutrality is the principal driver of the microbial community structure in urban and wild environments. To determine the strength of the neutral stochasticity, we utilized the Sloan neutral community model (NCM) (Sloan et al., 2006; Chen et al., 2019). This method evaluates the relationship between occupancy and species abundance, in which *R*^2^ indicates the overall fit to the neutral model (Sloan et al., 2006; Chen et al., 2019). In this model, the parameters Nm and *m* represent the metacommunity size times migration and migration rate, respectively, where higher m values indicate less dispersal limitation (Burns et al., 2016; Chen et al., 2019). To further assess the relative importance of stochastic versus deterministic processes in the community assembly, the phylogenetic normalized stochasticity ratio (pNST, ranging from 0 to 1) index was calculated using the *pNST* function in the ‘NST’ package in R (Ning et al., 2019, 2020). The pNST was used with 50% as the boundary point between stochastic assembly (>50%) and deterministic assembly (<50%). The total differences in the pNST between the U and W groups were tested by Kruskal–Wallis tests with multiple testing correction (Behrens–Fisher) using the *nmpc* function in the ‘nmpc’ R package (Munzel and Hothorn, 2001). In addition, the relationships between the pNST and urbanization, robustness and vulnerability were estimated with Spearman’s correlation analyses.

To evaluate the levels of OTU species co-occurrence patterns, the co-occurrence networks were constructed based on robust correlations with Spearman’s correlation coefficients (*r*) of >0.6 or <−0.6 and false discovery rate-corrected *p-*values of <0.01 using the ‘WGCNA’ R package (Langfelder and Horvath, 2008). To minimize the impacts of rare OTUs in the dataset, only OTUs with relative abundance >0.01% and the bacterial taxa that were detected in at least 10% of all samples per group were included in the analysis (Jiao et al., 2021). Subsequently, visualization of networks and the calculation of network properties were performed using the ‘igraph’ R package (Csardi and Nepusz, 2006). Additionally, we extracted the subnetwork topological characteristics of each sample by using the *subgraph* functions in the ‘igraph’ R package (Csardi and Nepusz, 2006; Ma et al., 2016). In this study, the network characterizations of the node numbers, edge numbers, average degree, degree centralization, graph density, average path length, clustering coefficient, graph modularity, and betweenness centralization were calculated to estimate the network structure differences in different groups. In addition, the robustness based on the method of 50% of nodes was randomly removed with 100 repetitions of the simulation, and the vulnerability was also calculated to assess the network stability (or ecological stability) for the symbiotic microbiota of amphibians from urban park and wild area habitats (Yuan et al., 2021; Liu et al., 2022). The total differences in the network characterization in different groups were tested by using Kruskal–Wallis tests with multiple testing correction (Behrens–Fisher) using the *nmpc* function in the ‘nmpc’ R package (Munzel and Hothorn, 2001). Furthermore, the relationships between the urbanization and robustness and vulnerability were assessed with Spearman’s correlation analysis.

To decipher changes in keystone taxa between Group 1 levels, we identified the putative role of each node based on two network topological characteristics of the within-module connectivity (*Zi*) and the among-module connectivity (*Pi*) following the previous classification: peripherals (*Zi* < 2.5 and *Pi* < 0.62), connectors (*Zi* < 2.5 and *Pi* > 0.62), module hubs (*Zi* > 2.5 and *Pi* < 0.62) and network hubs (*Zi* > 2.5 and *Pi* > 0.62) (Olesen et al., 2007). The connectors, module hubs and network hubs are generally defined as keystone taxa, which represent key species that may play crucial roles in maintaining the stability of the microbial community structure (Röttjers and Faust, 2019; Yuan et al., 2021; Liu et al., 2022).

### 2.3. Impacts of abiotic factors

We tested the impacts of abiotic variables on the microbial community structure and diversity. To do so, we utilized a set of variables, including 19 bioclimatic variables such as the latitude, longitude, urban_degree (degree of urbanization), elevation, PH, AT (air temperature) and AH (air humidity) (Supplementary Table S2). Bioclimatic variables were extracted from the WorldClim database.^2^ PH was measured using a portable HANNA HI 9913001/1 instrument, and AT and AH were detected every 10 min by using an automatic temperature and humidity recorder (Elitech GSP-8A) at each sampling site. In addition, we quantified the degree of urbanization by calculating the total proportion of three land use types, i.e., urban land (code 51), rural residential areas (52) and other construction land (53) to all land use classes within a 10-km radius buffer around each sampling site. Land use class data from 2020 (approximate to our sampling time) were obtained from the Resource and Environment Science and Data Center.^3^

Because the utilization of all variables could lead to multicollinearity, to reduce the risk of overfitting, we first utilized random forest (RF) to identify potentially highly correlated and important variables, which can indicate the importance of variables by the percentage increase in the MSE (mean squared error) (Jiao et al., 2021). In the RF models, the environmental variables are referred to as predictors for symbiotic microbial richness. Then, we selected the top 10 most important environmental variables for collinearity analysis using the *check_collinearity* function in the ‘performance’ R package (Lüdecke et al., 2021). Finally, we retained five environmental variables, the PH, AT, urban_degree, bio_3 (isothermality) and bio_18 (precipitation of warmest quarter) (Supplementary Table S2), for subsequent analysis. In this work, the relative abundance of the top 10 phyla and families and network characteristic correlations with environmental variables were calculated using the *corrplot* function in the ‘corrplot’ package (Wei et al., 2017). Moreover, redundancy analysis (RDA) was applied to disentangle the relationship between the symbiotic microbes of amphibians and environmental variables with the *rda* function of the ‘vegan’ package (Oksanen et al., 2002).

## 3. Results

### 3.1. Overview of the amphibian symbiotic bacterial composition and diversity

In terms of the community composition of host microbes, at the phylum level, for the skin and gut microbiota of amphibians, the bacterial composition was primarily dominated by Proteobacteria, Firmicutes, Actinobacteriota, and Bacteroidota. Specifically, for the skin microbiota of the three amphibians, the relative abundance of Proteobacteria, Firmicutes, and Actinobacteriota in wild areas (W_SK) was significantly higher than that in urban parks (U_SK) (Figure 1D). Conversely, for the gut microbiota, the relative abundance of Proteobacteria in wild areas (W_G) was significantly lower than that in urban parks (U_G), while the relative abundance of Firmicutes in the W_G group was significantly higher than that in the U_G group (Figure 1D). Furthermore, in contrast to the skin microbiota, Desulfobacterota and Verrucomicrobiota were also major phyla in the gut microbiota. Analogously, the relative abundance of Desulfobacterota in the W_G group was significantly higher than that in the U_G group, in addition to *Bufo gargarizans* (Figure 1D).

Interestingly, at the family level, the relative abundance of families in different groups presented remarkable microbial community composition differences (Figure 1D). For skin microbiota, Enterobacteriaceae, Comamonadaceae, and Burkholderiaceae were major dominant families. Additionally, in addition to Comamonadaceae and Burkholderiaceae, the relative abundance of Caulobacteraceae and Sphingomonadaceae of the three amphibians in the wild areas (W_SK) was also significantly higher than that in the urban parks (U_SK), whereas Enterobacteriacea had a higher relative abundance in the U_SK group (Figure 1D). For gut microbiota, Enterobacteriaceae was the dominant family in the urban parks (U_G), while Lachnospiraceae, Erysipelotrichaceae, Ruminococcaceae, and Desulfovibrionaceae were the major dominant families in the wild areas (W_G; Figure 1D).

**Figure 2:**
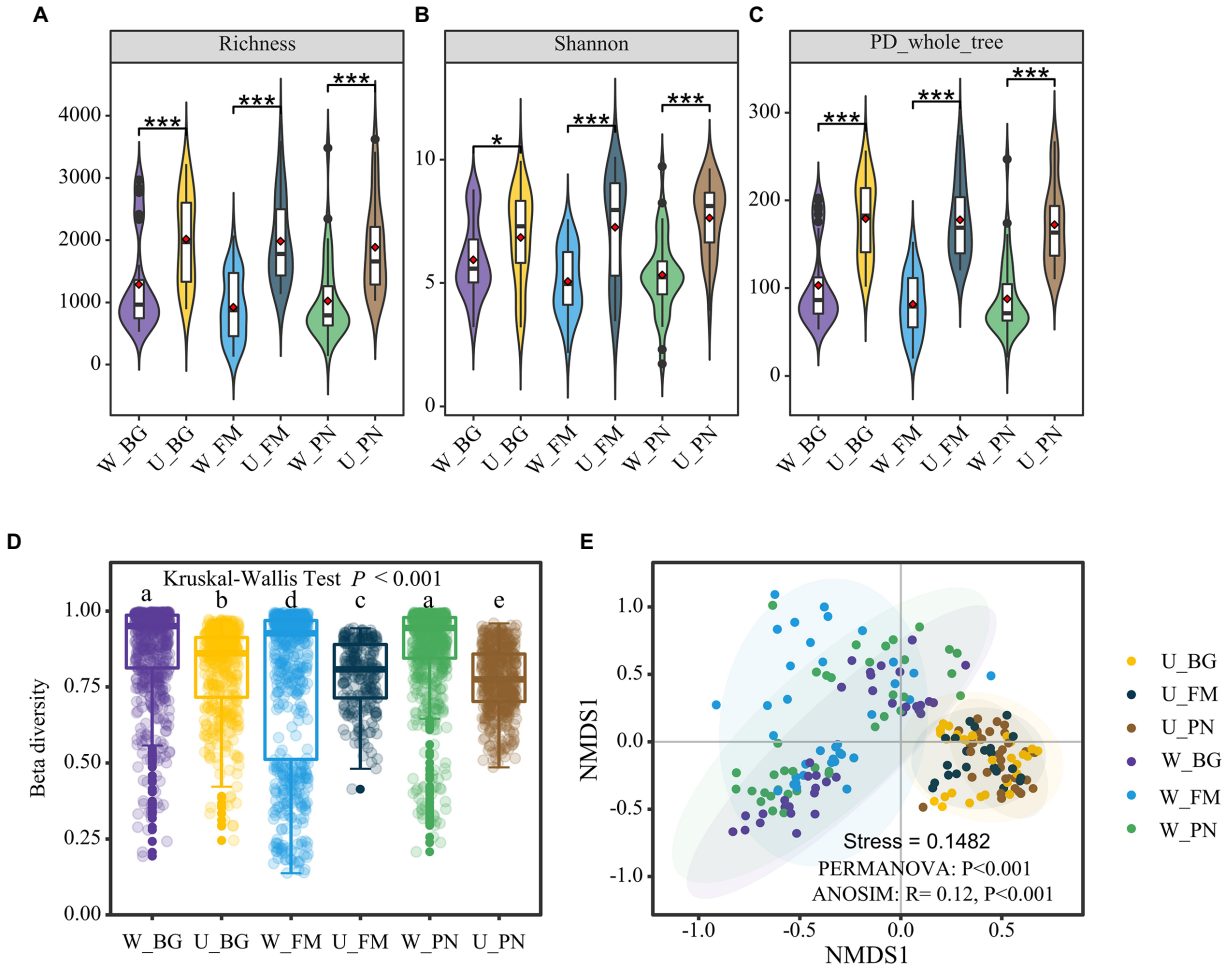
Microbial diversity of amphibians in the urban park and wild area habitats. Comparison of the alpha diversity between Group U (urban park samples) and Group W (wild area samples) amphibian symbiotic microorganisms, including the richness **(A)**, Shannon index **(B),** and PD phylogenetic diversity index **(C)**. The difference in the alpha diversity was tested using the Wilcoxon rank-sum test and false discovery rate (FDR) at the corrected level of *p* < 0.05. **(D)** Differences in the beta diversity between the U and W groups based on the Bray–Curtis distances. The different superscripts indicate significant differences between groups (*p* < 0.05, Kruskal–Wallis tests and Behrens–Fisher *post hoc* tests). **(E)** Nonmetric multidimensional scaling (NMDS) analysis based on the Bray–Curtis distance of the relative abundance of operational taxonomic units (OTUs). Differences are denoted as follows: ^∗^*p* < 0.05; ^∗∗^*p* < 0.01; ^∗∗∗^*p* < 0.001. W_BG, W_FM, and W_PN represent the symbiotic microbiota of *Bufo gargarizans*, *Fejervarya multistriata,* and *Pelophylax nigromaculatus* in the wild areas, respectively. U_BG, U_FM, and U_PN indicate the symbiotic microbiota of *Bufo gargarizans*, *Fejervarya multistriata,* and *Pelophylax nigromaculatus* in the urban parks, respectively.

In regard to the biological diversity of the microbes, at the alpha diversity level, the overall microbial diversity was consistently high in the U groups for the species-level richness (Figure 2A), Shannon index (Figure 2B), and phylogenetic diversity (Figure 2C). However, when comparing the alpha diversity difference for the skin and gut of host species, in general, skin microbes presented higher diversity (Supplementary Figure S2). This might be attributed to the fact that skin microbes are jointly influenced by host species and external abiotic environments. However, at the beta diversity level, the beta diversity built on the Bray-Curtis distance of the three amphibians was significantly higher in the W group than in the U group (Kruskal–Wallis tests, *p* < 0.001; Figure 2D; Supplementary Figure S3). Non-metric multidimensional scaling (NMDS) analysis was used to further disentangle this dissimilarity. The results show that there was clear segregation between the U and W groups, which presented significant differences (PERMANOVA, *p* < 0.001; ANOSIM, *R* = 0.12, *p* < 0.001; Figure 2E).

### 3.2. Urbanization influences the symbiotic bacterial community assembly of amphibians

**Figure 3:**
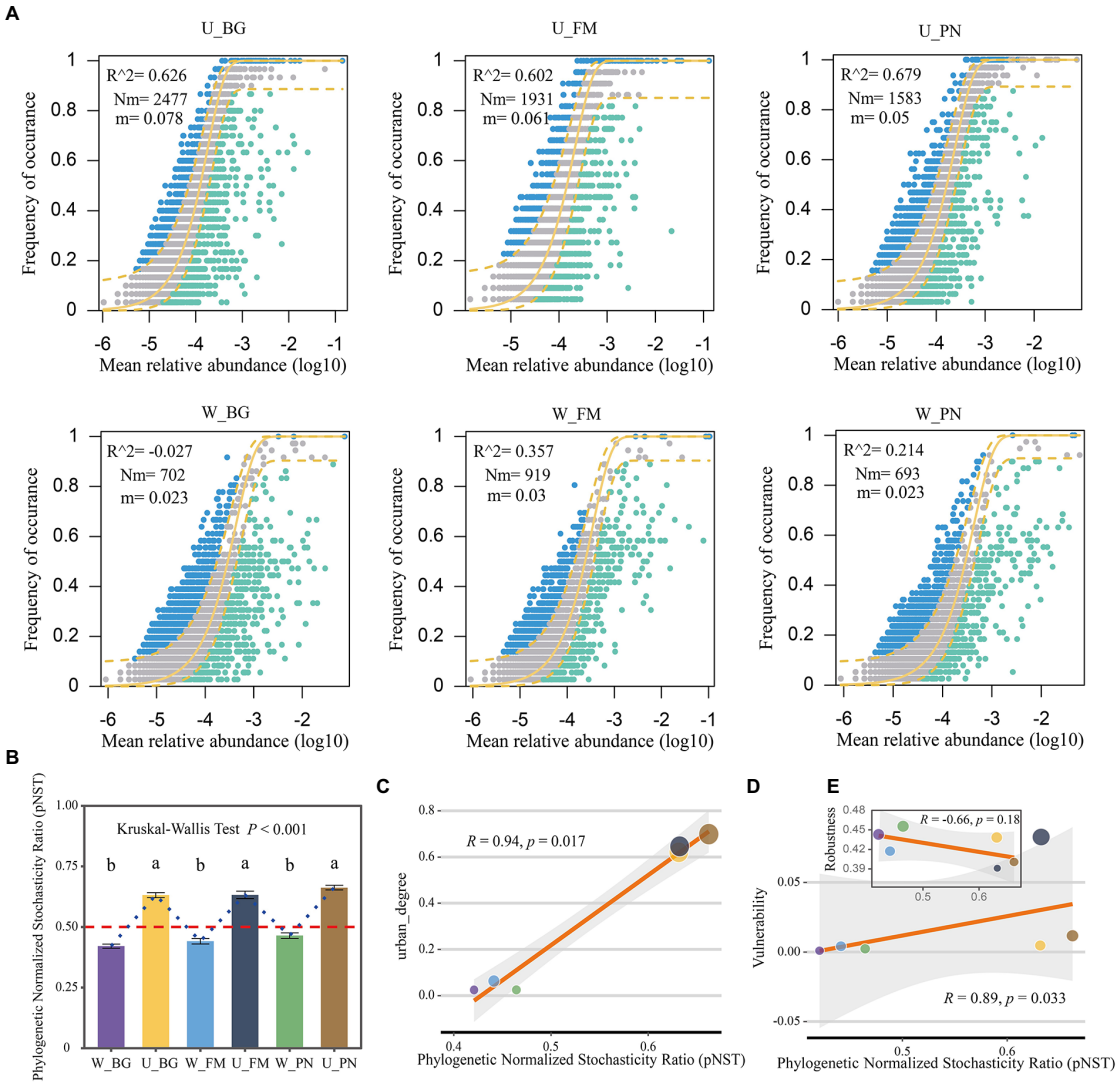
Microbial assembly mechanisms of amphibians in urban park and wild area habitats. **(A)** Neutral community model (NCM) applied to estimate the influence of random dispersal and ecological drift on the community assembly of the symbiotic microbiota of amphibians from urban parks and wild areas. Nm is the metacommunity size times immigration, *R*^2^ is the fit to the neutral model, and *m* indicates the migration rate. The solid yellow line indicates the best fit to the neutral model, while a dashed yellow line indicates 95% confidence intervals around the prediction. The different colors are used for OTUs that occur more or less frequently than predicted by the NCM. **(B)** The ecological stochasticity in the assembly of the amphibian symbiotic microbiota community evaluated by the phylogenetic normalized stochasticity ratio (pNST). The value of 0.5 is regarded as the boundary between a stochastic (>0.5) and deterministic (<0.5) assembly. The different superscripts indicate significant differences between groups (*p* < 0.05, Kruskal–Wallis tests and Behrens–Fisher *post hoc* tests). **(C)** Regression relationships between the pNST and urban_degree (urban degree of sampling sites). **(D,E)** Regression relationships between the pNST and the robustness and vulnerability, respectively. W_BG, W_FM, and W_PN represent the symbiotic microbiota of *Bufo gargarizans*, *Fejervarya multistriata,* and *Pelophylax nigromaculatus* in the wild areas, respectively. U_BG, U_FM and U_PN indicate the symbiotic microbiota of *Bufo gargarizans*, *Fejervarya multistriata,* and *Pelophylax nigromaculatus* in the urban parks, respectively.

Here, the NCM model was used to effectively evaluate the relationship between the occurrence frequency and relative abundance of OTUs. The results show that the explained proportion of variation (*R*^2^) of the symbiotic microbial community in the urban parks (U group: U_BG, *R*^2^ = 0.626; U_FM, *R*^2^ = 0.602; U_PN, *R*^2^ = 0.679) versus the wild areas (W group: W_BG, *R*^2^ = −0.027; W_FM, *R*^2^ = 0.357; W_PN, *R*^2^ = 0.214) present a remarkable difference. The greater *R*^2^ values of the U group indicate that stochastic processes played a more important role in the U group than in the W group (Figure 3A). This further suggests that urbanization enhanced the ecological stochasticity of the microbial community assembly. Furthermore, the Nm and m values were higher for microbial taxa in the U group (U_BG, Nm = 2,477, *m* = 0.078; U_FM, Nm = 1931, *m* = 0.061; U_PN, Nm = 1,583, *m* = 0.05) than in the W group (W_BG, Nm = 702, *m* = 0.023; W_FM, Nm = 919, *m* = 0.03; W_PN, Nm = 693, *m* = 0.023) (Figure 3A). These results illustrate that species dispersal of symbiotic microbes was higher in the U group than in the W group, indicating that the microbial taxa of the W group had a higher chance of dispersal limitation. Additionally, similar results were found in the gut and skin symbiotic microbe taxa between the U and W groups (Group 2 level) (Supplementary Figure S4).

The pNST model was further used to quantify the relative importance of stochastic and deterministic processes of the symbiotic microbe community assembly. The results show that the pNST value of the W group was <0.5, while that of the U group was >0.5, which indicates that stochastic processes and deterministic processes were the predominant processes for the W and U groups, respectively (Figure 3B). Moreover, significant differences in the pNST values were found between the U and W groups (Kruskal–Wallis tests, *p* < 0.001; Figure 3B). Importantly, the pNST was significantly positively correlated with urban_degree (Figure 3C), which further identifies the effect of urbanization on microbial community assembly processes. These results indicate that urbanization significantly alters microbial community assembly processes and increases stochastic processes in amphibian symbiotic microbes. These results are in line with previous NCM findings.

### 3.3. Urbanization influences the network structure of the amphibian symbiotic bacterial community

**Figure 4:**
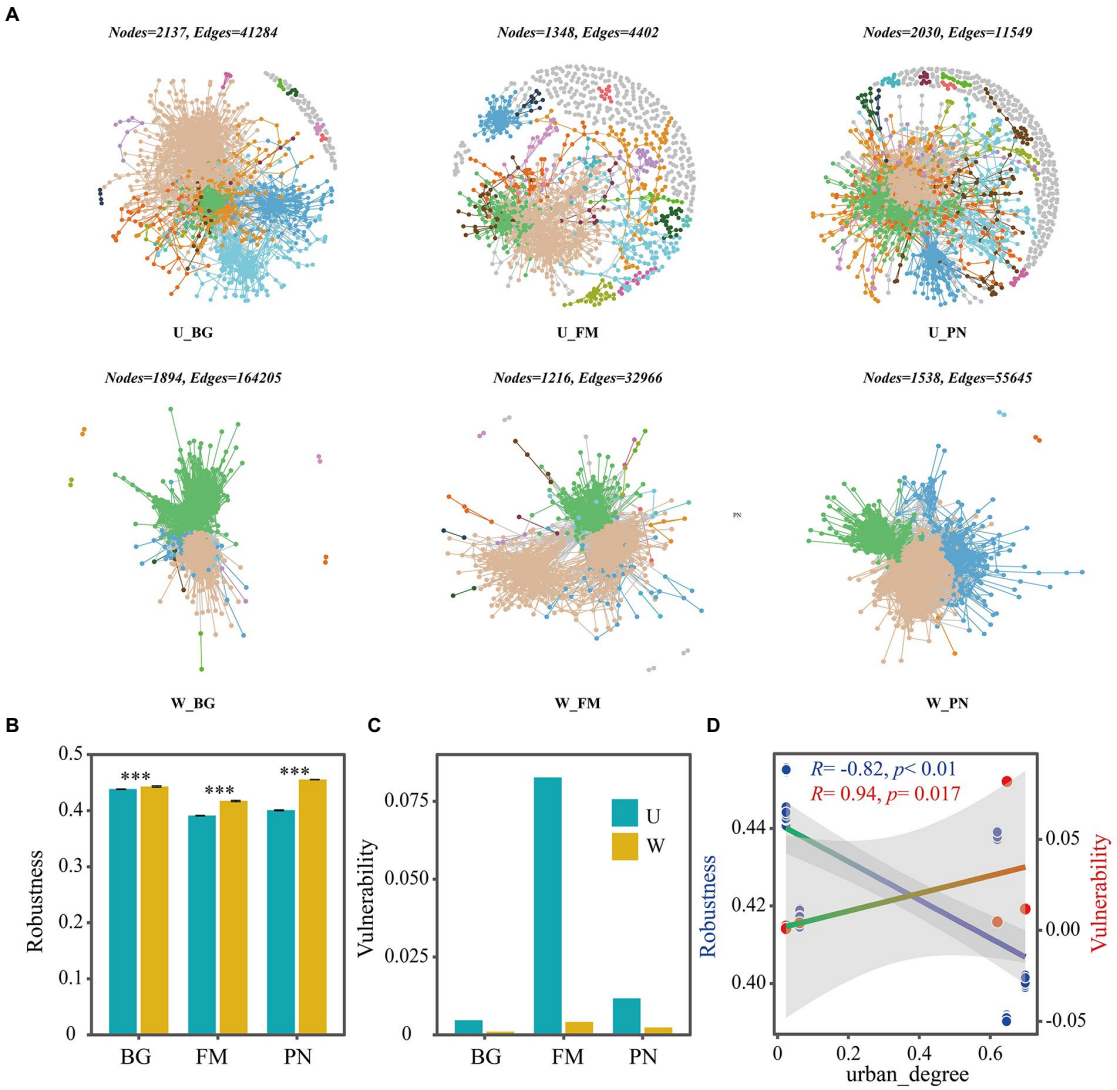
Co-occurrence networks of symbiotic microbial taxa of amphibians in the urban park and wild area habitats. **(A)** Network diagram of the symbiotic microbiota of amphibians in urban parks and wild areas. The robustness **(B)** was calculated as the proportion of the remaining species in the community after random node 50% removal, and the vulnerability **(C)** was determined by the maximum node vulnerability in each community. **(D)** The regression relationships between urban_degree (urban degree of sampling sites) and robustness and vulnerability. The difference in robustness was tested using the Wilcoxon rank-sum test and false discovery rate (FDR) at the level of corrected *p* < 0.05. Differences are denoted as follows: ^∗^*p* < 0.05; ^∗∗^*p* < 0.01; ^∗∗∗^*p* < 0.001. W_BG, W_FM, and W_PN represent the symbiotic microbiota of *Bufo gargarizans*, *Fejervarya multistriata,* and *Pelophylax nigromaculatus* in the wild areas, respectively. U_BG, U_FM, and U_PN indicate the symbiotic microbiota of *Bufo gargarizans*, *Fejervarya multistriata,* and *Pelophylax nigromaculatus* in the urban parks, respectively.

**Figure 5:**
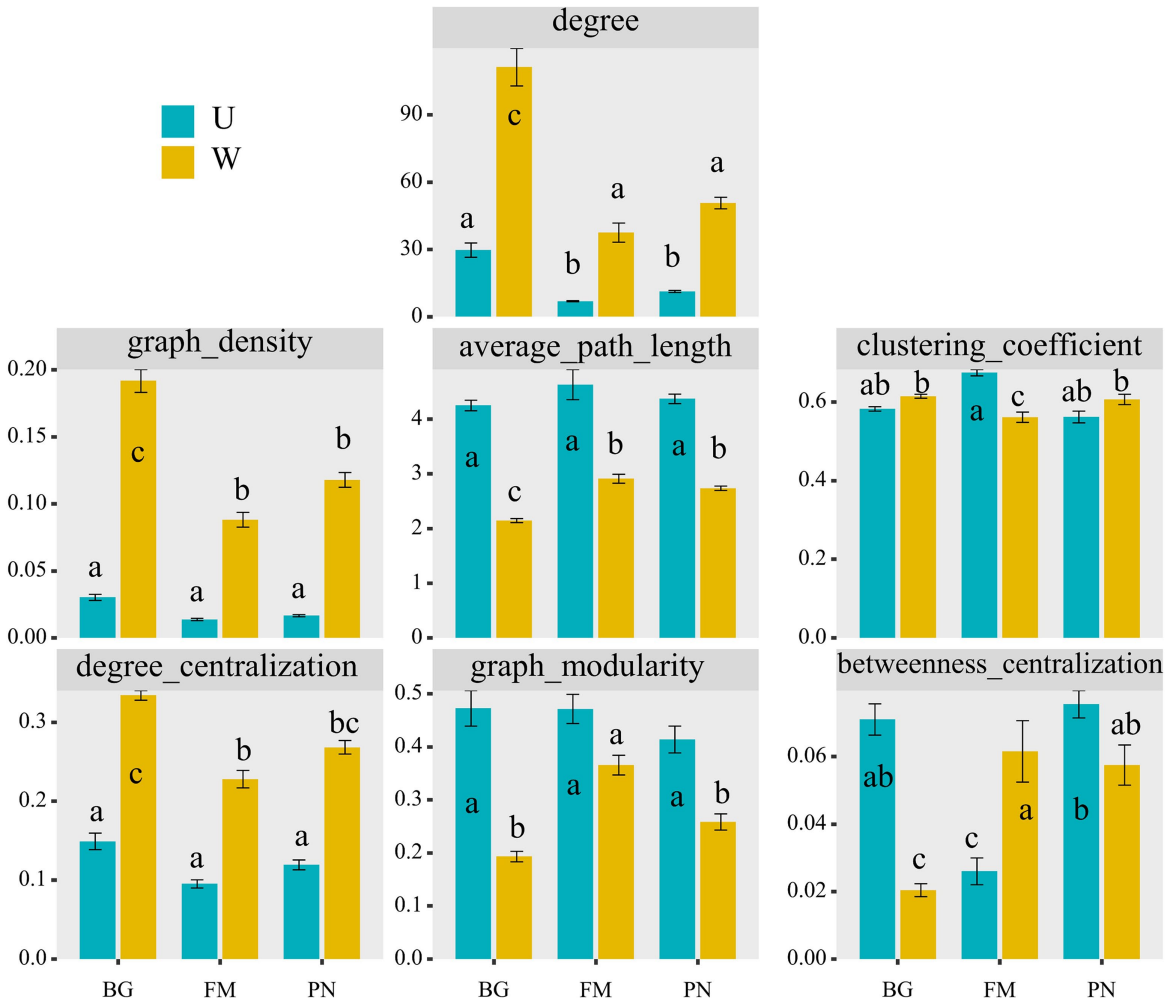
Comparison of different network characterizations between the U and W groups, including the node number (nodes_num), edge number (edges_num), average degree (degree), degree centralization, graph density, average path length, clustering coefficient, graph modularity, and betweenness centralization. The different superscripts indicate significant differences between groups (*p* < 0.05, Kruskal–Wallis tests and Behrens–Fisher *post hoc* tests). W_BG, W_FM, and W_PN represent the symbiotic microbiota of *Bufo gargarizans*, *Fejervarya multistriata,* and *Pelophylax nigromaculatus* in the wild areas, respectively. U_BG, U_FM, and U_PN indicate the symbiotic microbiota of *Bufo gargarizans*, *Fejervarya multistriata,* and *Pelophylax nigromaculatus* in the urban parks, respectively.

Using network analyses, we further identified important evidence for community simplicity, with a well-fitted power-law model (*R*^2^ = 0.995) for the degree distribution of the total network, which indicates that the network was scale-free and nonrandom (Barberan et al., 2012). The edge numbers were higher in the U groups than in the W group, whereas the node numbers were lower (Figure 4A). Importantly, the co-occurrence instances (edge/node ratio) in the U group (U_BG, edge/node = 19.32; U_FM, edge/node = 3.26; U_PN, edge/node = 5.97) were higher than those in the W group (W_BG, edge/node = 86.70; W_FM, edge/node = 27.11; W_PN, edge/node = 36.18), which indicates that the microbial taxa of the W group tend to co-occur more tightly and frequently (Figure 4A; Shu et al., 2021). The average degree and graph density, representing the network complexity (Wu et al., 2016), decrease from the W group to the U group, indicating that the W group had higher network complexity than the U group (Figure 5). The degree centralization shows similar patterns, suggesting that the network structures of the U group are more ‘even’ networks (Wu et al., 2016). Compared with the U group, the W group had a higher clustering coefficient and shorter average path length, indicating that the small-world properties of the W group were typical (Figure 5). Additionally, the betweenness centralization, which reflects the role of a node as a bridge between components of network (Banerjee et al., 2019), was higher in the U group than in the W group, excluding the FM amphibian (Figure 5).

As indicators of the network stability, the robustness and vulnerability (Yuan et al., 2021) were calculated. Our results show that the robustness was significantly higher (all *p* < 0.001) in the W group than in the U group (Figure 4B), while the vulnerability had the opposite trend (Figure 4C), suggesting that the U group had lower network stability than the W group. More importantly, the correlation results show that urbanization (urban_degree) was remarkably negatively correlated with the robustness (*R* = −0.82, *p* < 0.01), whereas vulnerability presented the opposite trend (*R* = 0.94, *p* = 0.017) (Figure 4D), which further suggests that urbanization significantly altered the robustness and vulnerability of the network. The above findings strongly indicate that urbanization profoundly affects microbial associations, significantly reduces complexity and stability, and enhances the vulnerability of the symbiotic microbial community.

**Figure 6:**
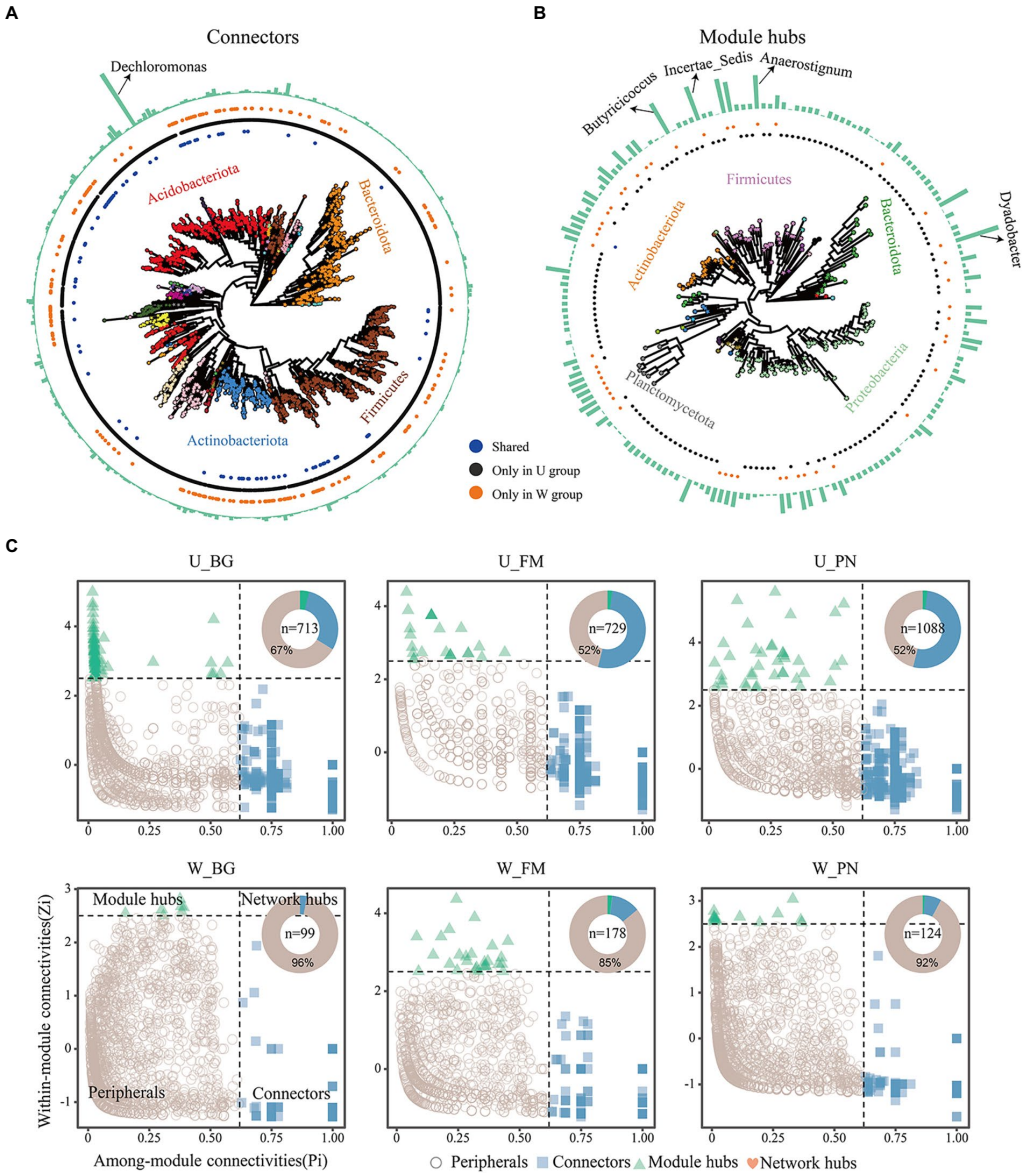
The keystone taxa distribution of amphibian symbiotic microbiota in urban parks and wild area habitats. The phylogenetic tree of keystone species of connectors **(A)** and module hubs **(B)**. Colours of the tip point indicate the phyla distribution. Blue, black, and orange dots indicate the keystone taxa that occurred in both urban and wild settings, only in urban setting (U group), and only in wild areas (W group), respectively. The outermost bar plot represents the degree of keystone taxa, and the annotations correspond to genus-level species. **(C)** Identifying keystone taxa across habitats based on their topological roles in networks. The pie chart shows the proportion of each keystone taxa. *n* denotes the number of keystone taxa, including the module hubs, network hubs, and connectors. W_BG, W_FM, and W_PN represent the symbiotic microbiota of *Bufo gargarizans*, *Fejervarya multistriata,* and *Pelophylax nigromaculatus* in the wild areas, respectively. U_BG, U_FM, and U_PN indicate the symbiotic microbiota of *Bufo gargarizans*, *Fejervarya multistriata,* and *Pelophylax nigromaculatus* in the urban parks, respectively.

The keystone taxa were identified to further estimate the effect of urbanization on the network structure of symbiotic microbes by using Zi–Pi relationships, with the connectors, module hubs and network hubs regarded as keystone taxa. The results reveal that the periphery had the most nodes in all groups, and the numbers of keystone taxa were higher in the U group (U_BG, *n* = 713; U_FM, *n* = 729; U_PN, *n* = 1,088) than in the W group (W_BG, *n* = 99; W_FM, *n* = 178; W_PN, *n* = 124) (Figures 6A–C), which demonstrates that urbanization notably increased the numbers of keystone taxa. Additionally, we found that the Acidobacteriota, Bacteroidota, Firmicutes, Actinobacteriota, Proteobacteria, and Planctomycetota play critical roles in maintaining the ecological stability of commensal microbes (Figures 6A,B). Dechloromonas at the genus level plays a dominant role in symbiotic microbes under urban settings (Figure 6A). Importantly, the degree of keystone taxa in group U was significantly lower than in group W, which may imply that many rare taxa play a more important role in regulating the ecological stability of symbiotic microbes in the urban setting (Figures 6A,B).

### 3.4. Impacts of urbanization and environmental variables on the symbiotic bacterial community of amphibians

**Figure 7:**
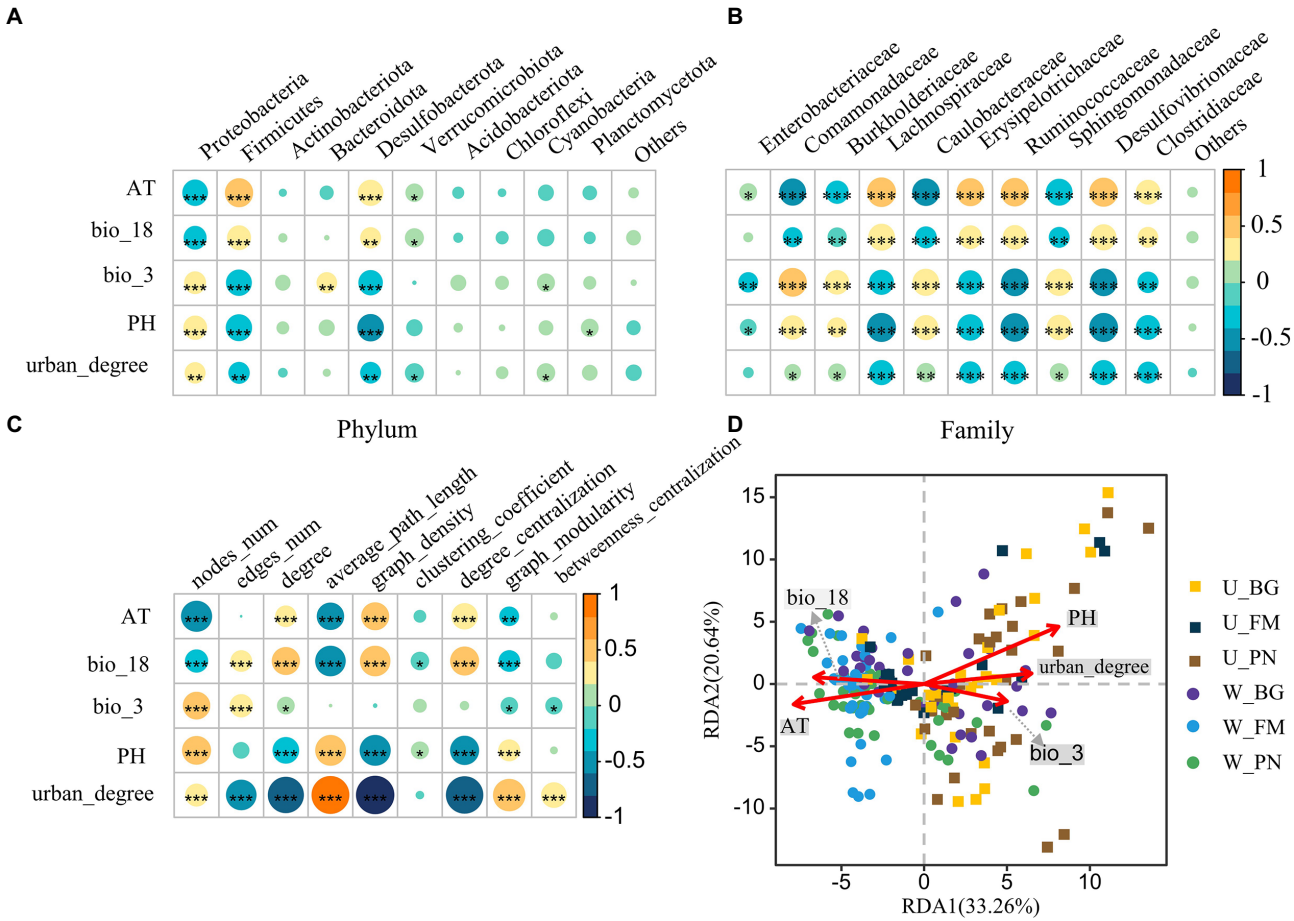
The relationship between the symbiotic microbes of amphibians and environmental variables. Correlations between environmental variables and the top 10 phyla **(A)** and families **(B)**. **(C)** Correlations between network characterizations and environmental variables. The size and color of the circles show the strength and sign of the correlation, respectively. Differences are denoted as follows: ^∗^*p* < 0.05; ^∗∗^*p* < 0.01; ^∗∗∗^*p* < 0.001. **(D)** Redundancy analysis (RDA) of the community structure of symbiotic microbiota amphibians and environmental variables, including bio_18 (precipitation of warmest quarter), bio_3 (isothermality), PH, AT (air temperature), and urban_degree (degree of urbanization). W_BG, W_FM, and W_PN represent the symbiotic microbiota of *Bufo gargarizans*, *Fejervarya multistriata,* and *Pelophylax nigromaculatus* in the wild areas, respectively. U_BG, U_FM, and U_PN indicate the symbiotic microbiota of *Bufo gargarizans*, *Fejervarya multistriata,* and *Pelophylax nigromaculatus* in the urban parks, respectively. The full names of the environmental factors are given in Supplementary Table S2.

The RF analysis show that bio_3, AT, urban_degree and PH were the four most important variables for symbiotic microbial richness (Supplementary Figure S1A). Specifically, correlations between the symbiotic bacterial composition and urbanization (urban_degree) and environmental factors were detected. At the phylum level (Figure 7A), the urban_degree, AT, bio_18, bio_3 and pH were significantly correlated (all *p* < 0.05) with the relative abundance of Proteobacteria, Firmicutes, and Desulfobacterota, respectively. At the family level (Figure 7B), the urban_degree and all variables presented significant correlations with the relative abundance of the top 10 families, excluding *Enterobacter australis*, for which urban_degree presented a remarkable positive correlation.

Additionally, we examined the correlations between the network topological features and environmental variables (Figure 7C). The results show that AT and bio_18 were significantly positively correlated with nodes_num, the average path length and graph modularity, and the correlations with the degree, graph density and degree centralization presented the opposite trend. Moreover, PH was negatively correlated with nodes_num, the average path length and graph modularity, while it was positively correlated with the degree, graph density and degree centralization. Importantly, significant positive correlations between the degree and graph density and urban_degree were observed, indicating that urbanization remarkably affects the complexity of microbial communities. Furthermore, correlations between urban_degree and edges_num and the degree centralization presented similar patterns, while urban_degree was significantly negatively correlated with nodes_num, the average path length, graph modularity and betweenness centralization.

The RDA further revealed the effect of urbanization and environmental variables on the symbiotic bacterial community (Figure 7D). The results show that five variables were correlated with the microbial community, especially PH, AT and urban_degree, with the RDA axis accounting for the total variance and RDA axis 2 accounting for 20.64%. The PH, urban_degree and bio_3 were positively correlated with the microbial community in the U group, while bio_18 and AT were negatively correlated. Moreover, the W group showed opposite correlations.

Finally, the relationship between the pNST and robustness was further identified. The results show that the pNST was remarkably positively correlated with the network vulnerability (*R* = 0.89, *p* = 0.033; Figure 3D), yet the robustness was slightly negatively correlated with the pNST (*R* = −0.66, *p* = 0.18; Figure 3E), which could indicate a certain degree of correlation between the stochasticity and network stability.

## 4. Discussion

The environmental disturbances, high spatial heterogeneity, and habitat fragmentation caused by urbanization (Gaston, 2010; Kondratyeva et al., 2020) can remarkably affect the composition and diversity of an animal’s symbiotic microbiome and adaption to complex urban settings (Lokmer et al., 2020; Barnes et al., 2021). In this study, we found that urbanization increased the richness and homogenization of microbes in amphibian hosts. First, in the alpha diversity analyses, we found that the microbial richness, abundance-based diversity (Shannon) and phylogenetic diversity tended to be higher in urban green spaces than in wild habitats (Figure 2A). This suggests that urbanization significantly increases the diversity of amphibian commensal microbes, while altering their kinship and phylogenetic diversity. Importantly, these results align with previous ecological theory that more diverse communities are resistant to invasion and further protect the host (Costello et al., 2012) as urban wildlife has more enhanced immune function, pathogen prevalence and stress than wildlife in wild areas (Murray et al., 2019). However, in our beta analyses, we found that the beta diversity of microbial taxa on the skin, in the gut or both organs generally presented a marked decrease in urban host specimens. Furthermore, ordination analyses (NMDS) showed that the microbial composition from urban samples tended to group together, but the microbial taxa from wild samples tended to be disperse in the ordination space. Therefore, it can be concluded that the microbial taxa of amphibian hosts presented high heterogeneity in wild areas and high homogeneity in urban areas. One explanation for this could be that the urban environments have similar local species pools and high dispersal rates, which drive the homogenization of symbiotic microorganisms (Figure 3A). This may suggest that there is remarkable habitat variation between sample sites in the wild, which leads to significant differentiation of symbiotic microorganisms. In contrast, urban habitats tend to have more similar habitats, which results in similar environmental pressures and symbiotic microbial convergence. More importantly, our results showed that a high alpha diversity does not necessarily imply high community compositional differences or high biotic heterogeneity for host-specific microorganisms.

Furthermore, we revealed the sensitivity and importance of amphibian skin as a first line of defense (Cramp et al., 2014; Xu et al., 2020a) in terms of community structural differences and community assembly. For instance, our results showed that the relative abundance of the remaining families (Figure 1D) and the three alpha indices (Supplementary Figure S2) of the skin microbes differed more between urban settings and wild areas than the gut microbes. At the community assembly (Supplementary Figure S4), we found that urbanization notably shifts differential patterns of stochasticity (*R*^2^) and dispersal rates (*m*) on the skin and gut symbiotic microbes. These results suggest that the environmental stressors imposed by urbanization markedly alter the conditions and niche spaces of the skin and gut, with the skin microbes becoming more sensitive (Xu et al., 2020a) and having a higher rate of dispersal and stochasticity versus the gut (Xu et al., 2022). Importantly, these findings will enhance our understanding of the mechanisms underlying the effects of urbanization on amphibians and contribute to the conservation of sensitive taxa.

Previous studies have revealed that stochastic processes dominate the assembly of skin microbial communities of amphibians along land use gradients, especially drift (Barnes et al., 2021). The stochasticity includes three assembly processes: homogeneous dispersal (as high dispersal rates can homogenize communities and cause high taxonomic turnover), dispersal limitation (as low dispersal rates could enhance community taxonomic alters) and ecological drift (Stegen et al., 2015; Ning et al., 2020). In this study, we found that urbanization increased the microbial stochasticity of amphibian hosts by transforming the dominant deterministic processes in wild areas into dominant stochastic processes in urban settings (Figure 3B). This change could be attributed to higher heterogeneity of conditions and the environmental filtering ability of different sampling sites in wild areas versus urban parks, while the sampling sites in urban parks have similar environmental stress and more numbers of rare or keystone taxa (Figure 6C). Interestingly, our results also verified that the amphibians in urban settings had lower abundance of existence of transient unique or rare symbiotic microbial taxa, such as more network nodes (Figures 4A, 5) or keystone taxa (Figures 6A,B) with no or low degree and a higher proportion of the remaining other families with lower abundance (Figure 1D).

The urban habitat is a separate local species pool, containing many new or unique microbial taxa, which provides a greater diversity of symbiotic taxa for the animals that live there (Barnes et al., 2021; Dillard et al., 2022). Previous studies show that rare taxa are susceptible to local exclusion by drift or competition with other dominant taxa (Melbourne and Hastings, 2008). However, in this study, the passive dispersal could play crucial roles in community assembly (Ai et al., 2013; Jia et al., 2018), as the diverse and distinctive urban local species spool may bring more low-abundance rare or unique taxa to the amphibians, thus causing high turnover and increasing the stochasticity (Stegen et al., 2015). These results align with the previous studies in which the assembly of rare species is mediated by stochastic processes (Jia et al., 2018). More importantly, this is further corroborated by our results that the symbiotic microbiome in urban parks has higher dispersal rates, indicating that homogeneous dispersal can play a profound role in the community assembly in urban settings. This may be due to frequent disturbances (such as human activities) and highly connected river network systems (Fan et al., 2022), which increase the exposure of amphibians to environmental microbes (especially water microbes) and could further increase dispersal rates. However, this may also introduce more new microbial taxa, thereby increasing the speciation, diversification, priority effects and colonization of new species, which may increase stochasticity (such as drift or homogeneous dispersal) (Vellend, 2010; Zhou and Ning, 2017).

In this study, stochastic features were also indicated by the random combination of occasional unique or rare microbial taxa at the OTU level in different locations and host individuals in urban samples, which is indicated by the phylum-and family-level compositional analyses of OTUs and the network analyses (Figures 4A, 6A,B). These microbial taxa increased the node number in the networks in urban areas, but they had few edges connecting other taxa in the networks (Figures 6A,B). Furthermore, although the average degree and graph density representing the network complexity over the urbanization gradient were markedly reduced, the network modularity, representing functional units (Luo et al., 2006) or niches (Eiler et al., 2012), and keystone taxa numbers exhibited an increasing trend (Figure 6C). This suggests that although urbanization reduces the complexity of the network, it increases the functional units to cope with various risks or pressures brought by the urban environment.

Urbanization can significantly change the ecological stability and complexity of environmental microorganisms (Yuan et al., 2021), but the impact on the ecological stability of symbiotic microorganisms has not been studied, which may be because it is a more complex triangular relationship involving the host, symbiotic microorganisms and environment. Here, we examine the network stability and complexity of a symbiotic community by calculating the robustness, vulnerability and topological properties. We found that urbanization reduces the network complexity and ecological stability, which may be one of the reasons for the increased randomness, since the network structure of microorganisms generally represents a nonrandom association (Barberan et al., 2012).

The key is that urban ecosystems are always disturbed and manipulated by human activities and pollution (Zhai et al., 2020; Yang, Y. et al., 2022). For the host species, they are completely different from wild habitats. For example, water bodies in city rivers, ditches or ponds are usually polluted by sewage emissions. Moreover, they are typically managed by local administrative units for the purpose of improving water quality. As such, microhabitat conditions are easily disturbed or altered because of human interference. Two typical pieces of evidence are the remarkable abiotic factor differences in water bodies and air features of urban settings: PH and AT showed significant correlations with symbiotic microbial composition and network topological properties. Additionally, we found that bio_18 and bio_3 also presented remarkable correlations. In summary, these results suggest the important roles of current and historical environmental factors in driving the dissimilarity of symbiotic communities between urban and wild settings.

More importantly, we quantified the degree of urbanization and revealed that it is significantly positively correlated with the stochasticity and vulnerability and significantly negatively correlated with the complexity and robustness. At the same time, we verified that the stochasticity has a significant negative correlation with the ecological stability, which is an interesting and important result. Consequently, our findings provide valuable and new insight into understanding the complex triangular relationship involving the host, environment, and symbiotic microbes.

It must be noted that there are some uncertainties (discussed in Supplementary Text S1 and Figure S5) in the analysis. For future research, it would be interesting to explore the impacts of urbanization on the host species community structure and how the impacts affect symbiotic microbiota. We argue that such analyses would clarify the triangular relationship between hosts, microbes and abiotic environments. In addition, it is important to test the transmission processes of microbial communities hosted within species at various trophic levels between food webs, which may have potential excavation value for understanding interspecific relationships in the context of global change. Furthermore, rare taxa play a pivotal role in regulating the community assembly and ecological stability of environmental microorganisms (Liang et al., 2020; Shu et al., 2021), but their specific regulatory mechanisms in amphibian symbiotic microbes along an urbanization gradient have not been quantitatively and deeply studied, especially the functional mechanisms and assembly processes of each taxon.

## Data availability statement

The data presented in the study are deposited in the National Center of Biotechnology Information (NCBI: https://www.ncbi.nlm.nih.gov/) repository, accession number PRJNA889361.

## Ethics statement

The animal study was reviewed and approved by the Animal Ethical and Welfare Committee of Chengdu Institute of Biology, Chinese Academy of Sciences.

## Author contributions

JZ analyzed the data, wrote the manuscript, performed the research, investigation, and methodology. ZiL wrote the manuscript. ZhL, XG, and WZ: investigation. YC: wrote the manuscript, methodology, and funding acquisition. All authors contributed to the article and approved the submitted version.

## Funding

This work was supported by the Strategic Priority Research Program of the Chinese Academy of Sciences (XDB31000000) and the Second Tibetan Plateau Scientific Expedition and Research Program, Grant Number 2019QZKK0303 the National Key Research and development Program of China (2020YFE0203200).

## Conflict of interest

The authors declare that the research was conducted in the absence of any commercial or financial relationships that could be construed as a potential conflict of interest.

## Publisher’s note

All claims expressed in this article are solely those of the authors and do not necessarily represent those of their affiliated organizations, or those of the publisher, the editors and the reviewers. Any product that may be evaluated in this article, or claim that may be made by its manufacturer, is not guaranteed or endorsed by the publisher.

## Footnotes

^1^ http://www.r-project.org/

^2^ https://www.worldclim.org/

^3^ https://www.resdc.cn/data.aspx?DATAID=335
