# Supplementary material for: Urbanization increases stochasticity and reduces the ecological stability of microbial communities in amphibian hosts
Source: Front Microbiol. 2023 Jan 12;13:1108662. doi: 10.3389/fmicb.2022.1108662 (PMC9878570; doi:10.3389/fmicb.2022.1108662)
Supplement: Supplementary file 1 [file Data_Sheet_1.pdf]

Supplementary Material

Urbanization increases stochasticity and reduces the ecological stability of microbial communities in amphibian hosts

Jin Zhou[1,2,3†], Ziyan Liao[1†], Zhidong Liu[1,3], Xuecheng Guo[1,3], Wenyan Zhang[1,3], Youhua Chen[1*]

*** Correspondence:** Corresponding Author: chenyh@cib.ac.cn

Supplementary Text S1

Examination of the impact of different sequencing equipment on the symbiotic microbial community structure.

In this study, we use two sequencing equipment (Miseq and Nova6000) to sequencing for amphibian symbiotic microbiome data, as some uncontrollable reasons. However, both Miseq and Nova6000 sequencing devices are part of the Illumina platform, so there is a high probability that there will be no significant batch effects. However, in this study, we also examine the potential influence of the involvement of different sequencing equipment on the community structure and diversity analyses of microbial taxa. The results showed that different sequencing machines did not have remarkably influenced the community structure and OTU classification (Fig. S5A, 5B). In addition, our unified standard data filtering of raw data also greatly reduces the impact of different sequencing equipment. Therefore, our study demonstrated that at least in our case, different sequencing equipment did not create dramatic differences on microbial community and diversity in amphibian hosts.

Supplementary Figures and Tables

FIGURE S1 Selection of important environmental factors. A. Potential environmental drivers for the richness difference of amphibian symbiotic microbes along urbanization gradient. Percentage increases in the MSE (%IncMSE) of variables were used to estimate the importance of these predictors, and higher %IncMSE values imply more important predictors. Differences are denoted as follows: *$p < 0.05$; **$p < 0.01$; ***$p < 0.001$; ns represent not significant. MSE = mean squared error; sig = significant environmental factors; In_sig = non-significant environmental factors. The representative meaning of 19 climate variables can be found on this website (https://www.worldclim.org/) and Table S2. B. Collinearity detection among environmental factors.

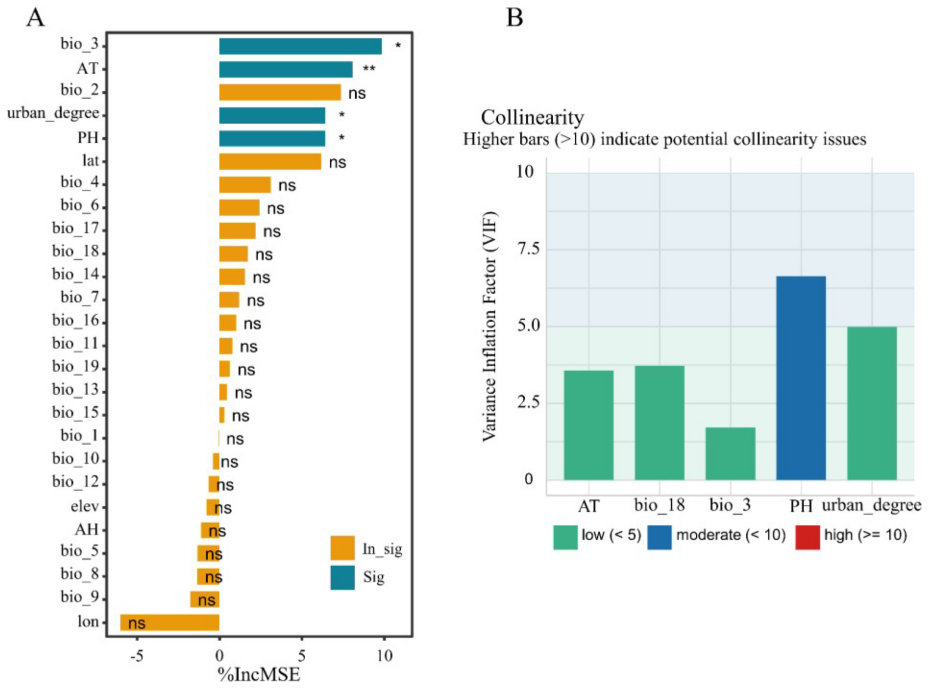

FIGURE S2 Microbial alpha diversity of different organs of amphibians in the urban parks and wild areas habitat. Comparison of alpha diversity between groups U (urban parks samples) and W (wild areas sample) of amphibian skin (abbr: SK) and gut (abbr: G) symbiotic microorganisms, including richness (A), Shannon index (B) and PD phylogenetic diversity index (C). The difference of alpha diversity was tested using Wilcoxon rank-sum test and false discovery rate (FDR) at the level of corrected p < 0.05. Differences are denoted as follows: *p < 0.05; **p< 0.01; ***p < 0.001; NS represent not significant. BG: *Bufo gargarizans*; FM: *Fejervarya multistriata*; PN: *Pelophylax nigromaculatus*. .

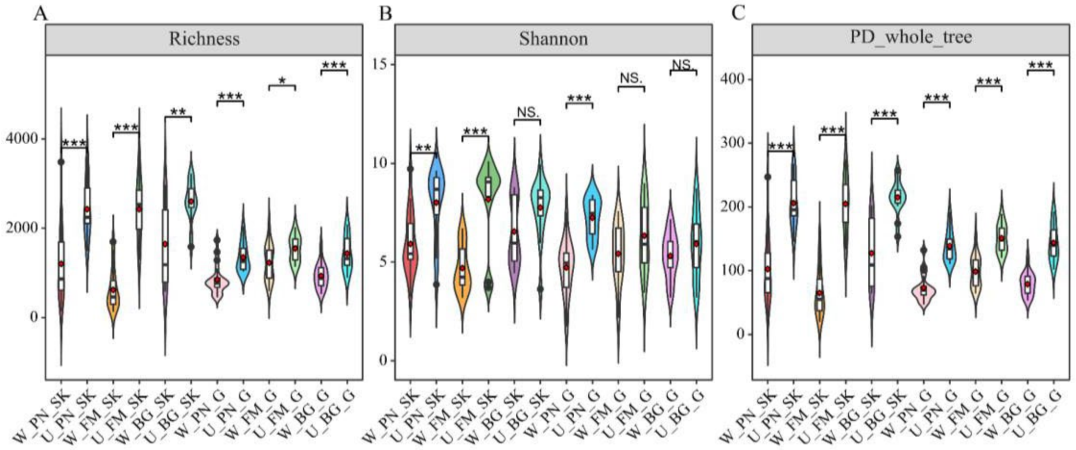

FIGURE S3 Microbial beta diversity of different organs of amphibians in the urban parks and wild areas habitat. Comparison of beta diversity between groups U (urban parks samples) and W (wild areas sample) of amphibian skin (abbr: SK) and gut (abbr: G) symbiotic microorganisms. The different superscripts indicate significant differences between groups ($p < 0.05$, Kruskal-Wallis tests and Behrens-Fisher *post-hoc* tests). BG: *Bufo gargarizans*; FM: *Fejervarya multistriata*; PN: *Pelophylax nigromaculatus*.

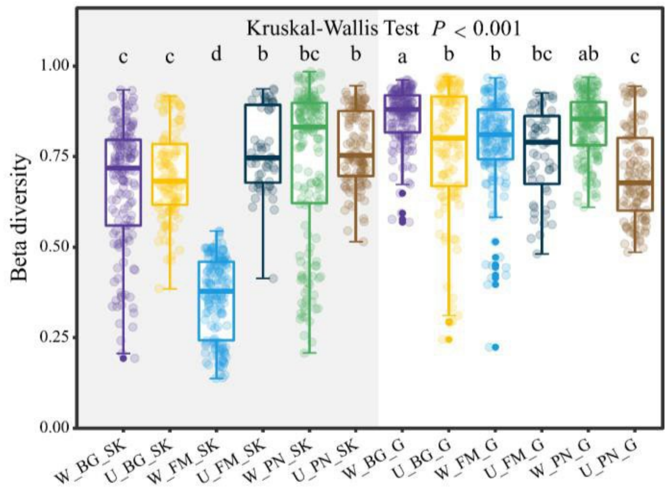

FIGURE S4 Microbial assembly mechanisms of different organs of amphibians in the urban parks (U group) and wild areas (W group) habitat. Neutral community model (NCM) applied to estimate the influence of random dispersal and ecological drift on the community assembly of skin (abbr: SK) and gut (abbr: G) symbiotic microbiota of amphibians from urban parks and wild areas. Nm exhibited the metacommunity size times immigration, R^2 exhibit the fit to the neutral model. The solid yellow line indicates the best fit to the neutral model, while a dashed yellow line indicates 95% confidence intervals around the prediction. The different color is used for OTUs that occur more or less frequently than predicted by the NCM. BG: *Bufo gargarizans*; FM: *Fejervarya multistriata*; PN: *Pelophylax nigromaculatus*.

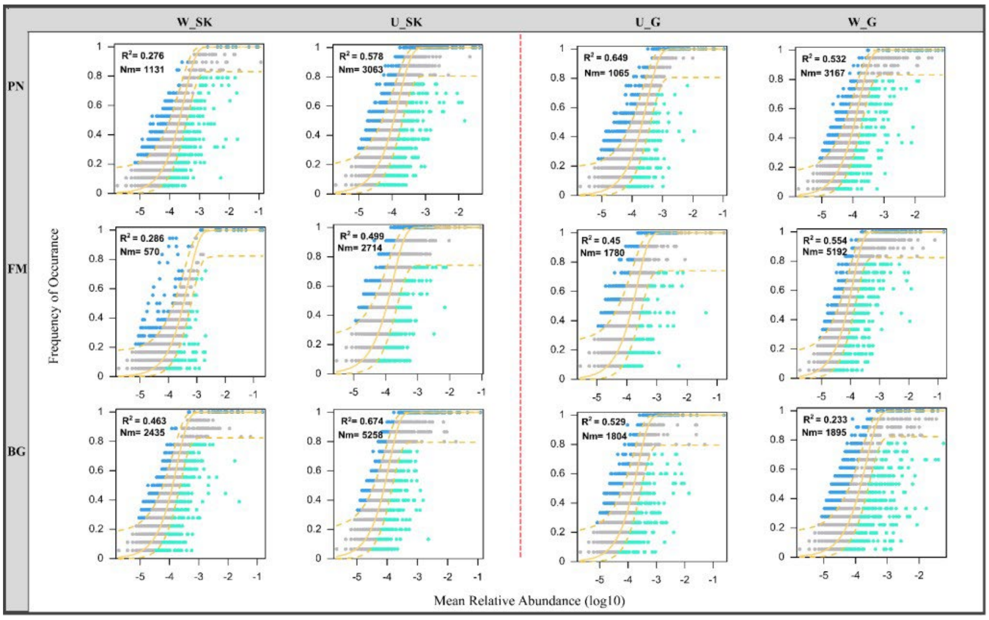

FIGURE S5 Detection of the impact of different sequencing equipment on the symbiotic microbial community structure. A. Bray-Curtis similarity-based dendrogram and composition stacked plot showing symbiotic bacterial community composition for all samples. B. Principal component analysis shows the effects of two sequencing equipment on symbiotic microbial community structure based on all symbiotic microbial data. MiSeq and Nova6000 represent the two the sequencing equipment of the Illumina platforms.

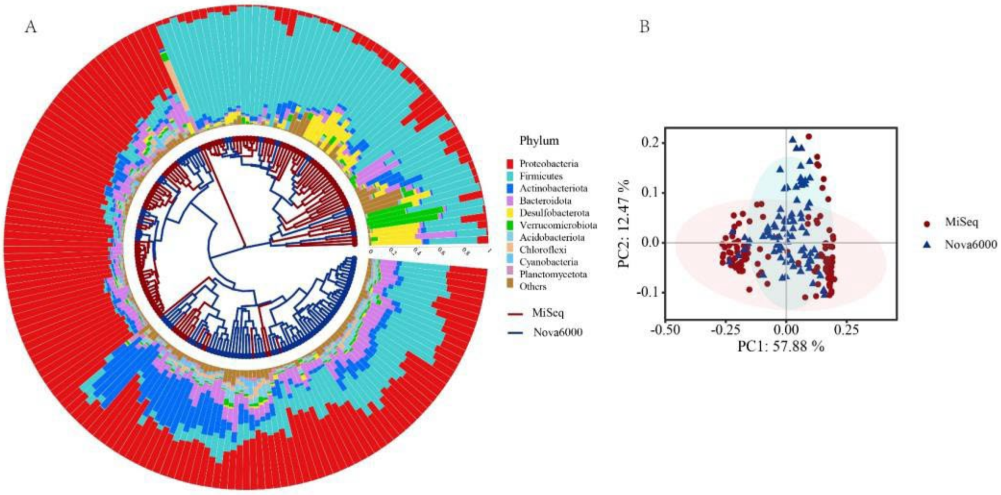

TABLE S1 Details of all sampling sites. N_{skin} and N_{gut} represent the number of skin and gut samples respectively. U and W denote the grouping of urban and wild samples, respectively.

Group	Sample sites	Longtitude	Latitude
W (N_{skin}=55; N_{gut}=55)	W1	104.35259	30.42695
	W2	104.05311	30.16083
	W3	103.94064	29.81665
	W4	103.90867	29.87228
	W5	103.69605	30.01412
	W6	103.23926	30.54053
	W7	103.21958	30.25115
	W8	102.67835	30.46461
U (N_{skin}=42; N_{gut}=42)	U1	104.08648	30.61833
	U2	104.07294	30.43686
	U3	104.05258	30.57301
	U4	104.10339	30.65855

TABLE S2 Abbreviations and full names of the environmental factors.

Abbreviation	Environmental factor (full name)
bio_1	Annual Mean Temperature
bio_2	Mean Diurnal Range (Mean of monthly (max temp - min temp))
bio_3	Isothermality (bio_2/bio_7) (×100)
bio_4	Temperature Seasonality (standard deviation ×100)
bio_5	Max Temperature of Warmest Month
bio_6	Min Temperature of Coldest Month
bio_7	Temperature Annual Range (bio_5-bio_6)
bio_8	Mean Temperature of Wettest Quarter
bio_9	Mean Temperature of Driest Quarter
bio_10	Mean Temperature of Warmest Quarter
bio_11	Mean Temperature of Coldest Quarter
bio_12	Annual Precipitation
bio_13	Precipitation of Wettest Month
bio_14	Precipitation of Driest Month
bio_15	Precipitation Seasonality (Coefficient of Variation)
bio_16	Precipitation of Wettest Quarter
bio_17	Precipitation of Driest Quarter
bio_18	Precipitation of Warmest Quarter
bio_19	Precipitation of Coldest Quarter
AT	Air temperature
AH	Air humidity
elev	Elevation
lat	Latitude
lon	Longtitude
urban_degre	Degree of urbanization
